# Entrectinib demonstrates prolonged efficacy in an adult case of radiation-refractory *NTRK* fusion glioblastoma

**DOI:** 10.1093/noajnl/vdac046

**Published:** 2022-04-13

**Authors:** Patrick T Grogan, Dustin A Deming, Jeffrey Helgager, Theresa Ruszkiewicz, Mustafa K Baskaya, Steven P Howard, H Ian Robins

**Affiliations:** Department of Medicine, Division of Hematology, Medical Oncology, and Palliative Care, University of Wisconsin, Madison, Wisconsin, USA; McArdle Laboratory for Cancer Research, Department of Oncology, University of Wisconsin, Madison, Wisconsin, USA; Carbone Comprehensive Cancer Center, University of Wisconsin, Madison, Wisconsin, USA; Department of Medicine, Division of Hematology, Medical Oncology, and Palliative Care, University of Wisconsin, Madison, Wisconsin, USA; McArdle Laboratory for Cancer Research, Department of Oncology, University of Wisconsin, Madison, Wisconsin, USA; Carbone Comprehensive Cancer Center, University of Wisconsin, Madison, Wisconsin, USA; Department of Pathology and Laboratory Medicine, University of Wisconsin, Madison, Wisconsin, USA; Department of Human Oncology, University of Wisconsin, Madison, Wisconsin, USA; Department of Neurological Surgery, University of Wisconsin, Madison, Wisconsin, USA; Department of Human Oncology, University of Wisconsin, Madison, Wisconsin, USA; Department of Medicine, Division of Hematology, Medical Oncology, and Palliative Care, University of Wisconsin, Madison, Wisconsin, USA; Carbone Comprehensive Cancer Center, University of Wisconsin, Madison, Wisconsin, USA; Department of Human Oncology, University of Wisconsin, Madison, Wisconsin, USA; Department of Neurology, University of Wisconsin, Madison, Wisconsin, USA

The National Comprehensive Cancer Network (NCCN) guidelines for glioma in 2021 outline larotrectinib and entrectinib as appropriate therapies for disease harboring neurotrophic tyrosine kinase (*NTRK*) gene fusions.^1^ While some efficacy has been reported with larotrectinib in case reports and early clinical studies, to date, there have been no published reports of the successful use of entrectinib in adult gliomas, including glioblastoma (GBM). Here, we outline the first known case of successful use of this agent in the recurrent setting for an adult patient with an *NTRK* fusion (*BCR-NTRK2*) GBM.

The patient, at diagnosis, was a 67-year-old male with a medical history of atrioventricular block with syncope status post-pacemaker placement, paroxysmal atrial fibrillation, and benign prostatic hypertrophy. He presented to the emergency department with confusion in the setting of several weeks of headaches and subjective agitation, memory loss, and fatigue. CT angiography in the setting of concern for a stroke revealed a heterogeneously enhancing, centrally necrotic mass right temporal lobe. Subsequent MRI confirmed a 6.9 × 4.5 × 5.3 cm mixed cystic and solid mass showing restricted diffusion and extensive T2/FLAIR signal consistent with a GBM (Figure 1A).

**Figure 1.**
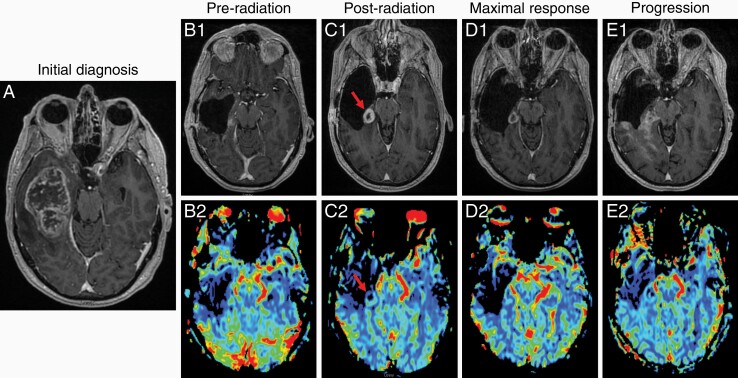
T1-weighted contrasted MRI sequence of the patient’s GBM at the time of diagnosis (A). T1-weighted contrasted sequences (1) and relative cerebral blood volume sequences showing perfusion (2) on MRIs 1 week prior to radiation therapy (B), surveillance 8 weeks after radiation showing disease recurrence (red arrows) with associated perfusion (C), 33 weeks after the initiation of entrectinib where maximal response to therapy was observed (D), and 15 months after entrectinib initiation where disease progression was observed (not all sites of disease pictured). Not pictured here is further rapid, extensive bihemispheric symptomatic progression captured on MRI 15 days after the initial identification of progression. Abbreviations: GBM, glioblastoma; MRI, magnetic resonance imaging.

The day after the presentation, the patient underwent a right frontotemporal craniotomy with gross total resection. Pathology returned a diagnosis of GBM, *IDH*-wildtype, WHO grade 4 (Figure 2A–C). *IDH1* and *IDH2* genes were confirmed wildtype by PCR, and *MGMT* promoter was unmethylated (methylation score 0.14; positive cutoff >2) by Labcorp methylation-specific PCR. He was discharged to inpatient rehabilitation and made a full functional recovery postoperatively.

**Figure 2.**
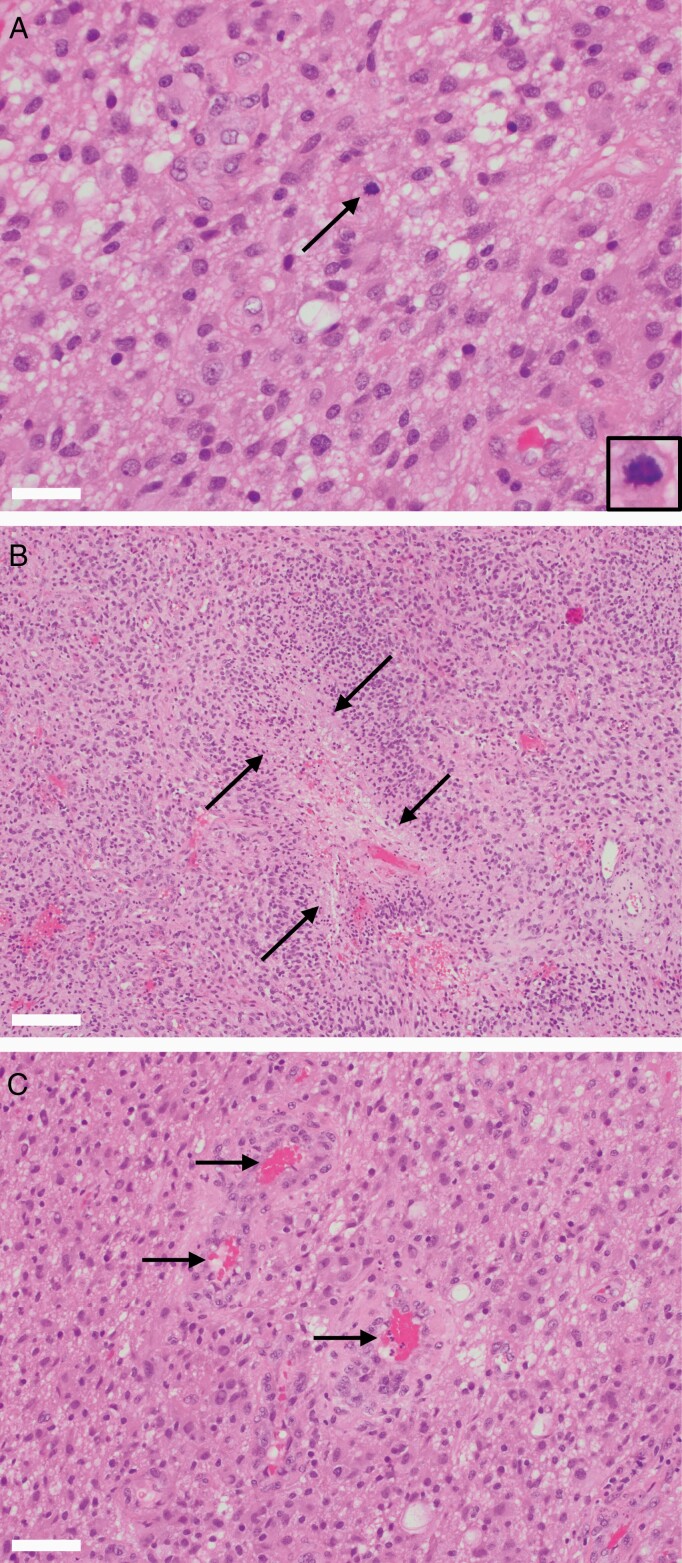
Histology of the patient’s glioblastoma, *IDH*-wildtype. Hematoxylin and eosin (H&E)-stained sections demonstrated a glial neoplasm with a fibrillary background and moderate-to-severe cellularity and nuclear atypia (A), as well as scattered mitoses (inset, circle). Necrosis, frequently palisading (B, arrows), was abundant with multiple foci of microvascular proliferation (C, arrows). Notably, the histology was within the spectrum of that seen in *IDH*-wildtype glioblastoma, with no obvious unusual features that may correlate with the presence of a unique molecular fusion event. Scale bars = 50 mm (A), 200 mm (B), 100 mm (C). Abbreviation: *IDH*, isocitrate dehydrogenase.

Comprehensive targeted tissue genomic profiling was completed with the StrataNGS platform (Strata Oncology, Ann Arbor, MI, USA). This revealed the well-characterized *TERT* C228T promoter mutation, supporting the diagnosis of *IDH*-wildtype GBM, and a *KMT2C* G3796S variant of unknown significance. Surprisingly, however, there was also a *BCR-NTRK2* fusion with exon 1 of the breakpoint cluster region gene (*BCR*; transcript NM_021574.3) fused to exon 17 of *NTRK2* (transcript NM_006180) (Figure 3). There were no noted alterations in *CDKN2A/B*, *EGFR*, *CDK4/6*, or *PTEN*, and the tumor mutational burden was 2 mutations per megabase, while StrataNGS is not formally validated to assess single-copy changes, one copy gain of chromosome 7 and one copy loss of chromosome 10 were observed. We engaged in extensive discussions about the use of radiation, temozolomide (TMZ), and tumor-treating fields (TTF) as standards of care including the limited benefit of TMZ in the setting of an *MGMT*-unmethylated GBM and the potential risks given his historical periods of lymphopenia.

**Figure 3.**
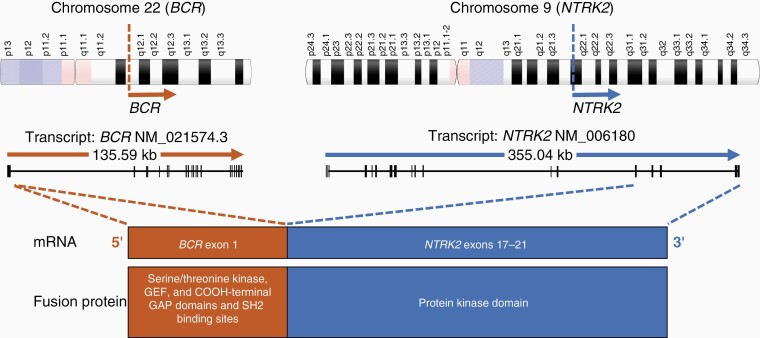
Illustrative representation of the *BCR-NTRK2* fusion as identified by StrataNGS on the patient’s resected GBM tissue. Location of the *NTRK2* and *BCR* genes on chromosomes 9 and 22, respectively. Sequencing showed fusion of exon 1 of BCR from reference transcript NM_021574.3 to exon 17 of NTRK2 from reference transcript NM_006180. The protein kinase domain of NTRK2 was retained with exons 17-21. In the patient’s sample, the BCR-NTRK2 fusion was detected by 98 824 reads on multiplex PCR-based StrataNGS RNA sequencing (0.2% of 4 539 742 total mapped reads). Minimum acceptance criteria for the gene fusion component of StrataNGS is >50 000 total mapped reads with reporting of individual fusion isoforms if greater than 1000 transcripts are present. Abbreviations: *BCR*, breakpoint cluster region protein; GBM, glioblastoma; *NTRK2*, neurotrophic receptor tyrosine kinase 2.

Given the *NTRK2* fusion and his age as well as quality of life considerations, the patient opted to move forward with hypofractionated radiation (40.05 Gy in 15 fractions) without chemotherapy or TTF. This was initiated approximately 7-week post-surgery with a delay secondary to pacemaker coordination issues. A pre-radiation MRI demonstrated postoperative change and no convincing evidence of interval tumor regrowth (Figure 1B1 and B2). Eight weeks after the completion of radiation, the patient underwent a surveillance MRI which demonstrated interval development of a thick rim enhancing lesion (21.8 × 14.5 × 15.7 mm) posteromedially to the resection cavity with associated increased T2/FLAIR, new perfusion, and diffusion restriction consistent with rapidly recurrent disease rather than pseudoprogression (Figure 1C1 and C2). Given the location of the lesion relative to the hippocampus, there was concern regarding the potential for quality of life implications if a re-resection was completed.

As such, *NTRK* fusion-targeted therapy was recommended with the support of the University of Wisconsin Precision Medicine Molecular Tumor Board, particularly in light of a lack of other classic concurrent driver alterations for a GBM. Entrectinib, an *NTRK*/*ROS1*/*ALK* tyrosine kinase inhibitor, was generously provided by Genentech through a compassionate use protocol, and the patient was initiated on these 10 days after the radiographic evidence of recurrence. Subsequent MRIs demonstrated slowly decreasing size of the recurrent lesion with maximal response (13.7 × 10.6 × 11.3 mm; 66.3% reduction by enhancing volume) obtained 33 weeks after treatment initiation (Figure 1D1 and D2) with stable disease until the time of progression. Entrectinib was well tolerated with mild anemia and lymphopenia as well as grade 1-2 creatinine elevation that resolved with dose reduction, ultimately from 600 mg to 400 mg daily approximately 4 months after starting the drug. Given the patient’s cardiac history, interval monitoring through electrocardiography and echocardiography was performed with no changes noted.

Notably, approximately 15 months after the initiation of entrectinib, the patient remained asymptomatic but underwent a surveillance MRI that demonstrated GBM progression with new and extensive contrast enhancement along the medial margin of the right lateral ventricle and right parietal white matter as well as extension into the corpus callosum (Figure 1E1 and E2); increased T2/FLAIR hyperintensity corresponded to these regions only. Two weeks later, the patient had a dramatic further clinical deterioration requiring hospitalization for intermittent confusion, nausea, and generalized weakness with inability to effectively ambulate. An MRI was repeated and demonstrated a significant increase in enhancing tumor throughout the right temporal lobe extending into the parietal lobe, occipital lobe, and across the splenium as well as the right fornix and ependymal surface of the right lateral ventricle. These changes were associated with restricted diffusion, increased cerebral blood volume, and minimal edema on T2/FLAIR series. Relative to imaging 15 days before, the volume doubling time, as measured in different sites of the nonlinear T1 contrast-enhancing lesions, was between 4 and 9 days, a rapid escape from prior entrectinib-maintained disease control.

To our knowledge, this is the first report of entrectinib used in an adult glioma, either successfully or unsuccessfully. The NCCN guidelines for adult glioma have recently evolved to incorporate molecular alterations now identified through expanding use of next-generation sequencing (NGS) techniques.^1^ These specifically outline *NTRK* fusions and the *BRAF* V600E mutation as alterations that are potentially actionable with targeted therapy. Although NGS had not historically been routinely utilized in high-grade gliomas, these guideline updates have led to improved insurance acceptance and coverage for such testing as a standard of care.

Given the lack of meaningful overall survival benefits with approved therapies in the recurrent GBM setting (CCNU, bevacizumab, TMZ, regorafenib, PCV chemotherapy), novel therapies are desperately needed in the management of this disease. Genomic sequencing potentially introduces targeted therapy options for certain patients in the recurrent setting. Emerging reports have identified a substantial number of GBMs that respond favorably to genomically informed on- and off-label precision-based therapy.^2^ Given failures of PI3K and CDK4/6 inhibitors among others, the use of such agents will need to be done with cautious optimism.^3–5^ Gene fusions in glioma necessitate the use of NGS platforms that incorporate not only DNA profiling but also RNA sequencing for transcriptome identification. The molecular profile of our patient’s GBM was unique given the lack of other frequently observed concurrent driver alterations, making NTRK-targeted therapy more appealing.

*NTRK* gene fusions in glioma are rare, representing <2% of cases at most with variable incidence among infantile, pediatric, and adult populations.^6^ FDA approval was provided for larotrectinib and entrectinib in *NTRK* fusion-positive cancers based on promising phase 1/2 studies.^7,8^ The entrectinib studies, while not enrolling adult primary central nervous system (CNS) tumor patients, showed a 50% response rate (n = 12) in patients with CNS metastases with preclinical data similarly showing entrectinib’s ability to cross into the brain. A pooled analysis of larotrectinib treatment for primary CNS malignancies from the SCOUT (NCT02637687) and NAVIGATE (NCT02576431) trials along with case reports have shown activity of larotrectinib against primary CNS disease including *NTRK* fusion glioma with understandably limited patient numbers.^9–12^ The pooled analysis, which consisted of 33 total participants, 78% of whom were pediatric patients, demonstrated a 30% overall response rate, less than the 78% seen in non-CNS disease. The 7 adult patients all had high-grade gliomas and ranged from 26 to 78 years old with all but one of the fusions involving *NTRK2*. Six of the patients achieved stable disease based on RANO criteria with the other one progressing. However, of 6 patients for which lesion size could be quantifiably assessed, 4 achieved a decrease in tumor size that did not qualify as a RANO partial response, and the 24-week disease control rate was 57% in adult patients.^12^

Despite differing tumor behavior in younger patients compared to adults, entrectinib too has been successfully employed in small numbers of fusion-positive pediatric and infantile CNS tumors including gliomas per preliminary data from the STARTRK-NG.^13,14^ One patient with a similar *BCR-NTRK2* fusion was included in the STARTRK-NG data with stable disease achieved. In addition to glioma, this alteration has also been noted in a treatment-refractory pilocytic astrocytoma, though with fusion to exon 13 rather than 17 of the *NTRK2* gene.^15^

Management of GBM in adult patients remains challenging with well-known therapeutic and outcome limitations. Targeting alterations like NTRK fusion proteins, when possible, has shown clinical promise. It remains unclear which *NTRK* fusion partners or translocation sites provide more meaningful and/or actionable activation, if any, as well as the implications of frequently co-occurring genomic alterations that may provide escape pathways to NTRK-directed therapy. Concurrent alterations in *NTRK* fusion gliomas demonstrate increased frequency with older age.^6^ In a cohort of 22 adults patient with *NTRK* fusion glioma, the most frequently detected concurrent genomic changes included *CDKN2A/B* deep deletion, *TERT* promoter mutation, *PTEN* loss or mutation, *TP53* mutation, *IDH1* mutation, polysomy 7, and *RB1* loss or mutation, with most observed at frequencies consistent with that seen in GBM. Interestingly, amplification of the epidermal growth factor tyrosine kinase receptor (*EGFR*) gene was only seen in 2 patients, well below expected levels for GBM patients and perhaps reflective of the activation of the resultant NTRK fusion protein.^6^ Our patient was an ideal candidate for targeted therapy in the setting of limited concurrent alterations, a well-characterized activating partner in exon 1 of the *BCR* gene, and the *NTRK2* translocation region.^16^

Sampling of our patient’s tumor at the time of recurrence on entrectinib was not feasible due to declining clinical status. However, resistance to first-generation NTRK inhibitors has been well documented to occur through various mutations in the ATP-binding pocket of the kinase domain as well as off-target alterations including the mitogen-activated protein kinase (MAPK) pathway.^17,18^ Multiple next-generation NTRK inhibitors to overcome on-target resistance have been developed, and while these have been shown promise in the preclinical and early phase trial settings, robust in-human data are lacking.^17,19^ Clinical trials for selitrectinib (NCT03215511) and reporectinib (NCT03093116) in the *NTRK* fusion-positive cancers resistant to first-generation agents are ongoing.

This case provides the first reported successful but transitory use of entrectinib in an adult GBM patient and supports its inclusion alongside larotrectinib in the management of *NTRK* fusion-positive gliomas. It is important to note that, in contrast to fusions, preclinical data have demonstrated variable response rates of malignancies harboring *NTRK* mutations and amplifications to NTRK inhibition with subsequent clinical exploration yielding disappointing results. While the decision to initiate NTRK-targeted therapy shortly after the completion of frontline therapy was straightforward in the setting of rapid disease recurrence for our patient with disease harboring an *NTRK2* fusion, there are no data regarding appropriate timing in patients who have completed upfront therapy with subsequent disease stability. Current NCCN guidelines recommend the use of entrectinib and larotrectinib in the recurrent setting, though given the near-inevitability of disease recurrence in GBM and its rapid growth kinetics, some clinicians argue that initiation of therapy prior to disease progression in the adjuvant setting may be preferred. It remains unknown if earlier initiation of therapy delays radiographic or symptomatic recurrence/progression, improves long-term quality of life relative to immediate drug toxicity implications, extends overall survival, or would even be covered by standard health insurance policies. Given the rarity of these alterations in an already uncommon disease type, such implications are unlikely to ever be meaningfully explored. Therefore, the risks and benefits of each approach should be considered in individual circumstances and in the context of thorough informed consent.

## Notes

*Authorship statement.* Acquisition of material: P.T.G., M.K.B., and J.H. Direct patient management/data implementation: P.T.G., J.H., T.R., M.K.B., S.P.H., and H.I.R. Analysis of data/information: P.T.G., D.A.D., J.H., T.R., M.K.B., S.P.H., and H.I.R. Interpretation of data/information: P.T.G., D.A.D., J.H., and H.I.R.
